# Predictive factors of surgical difficulty in laparoscopic cholecystectomy in a secondary hospital

**DOI:** 10.1590/acb410126

**Published:** 2026-01-16

**Authors:** Victoria Casarim, Isabela Martins Soares Miranda, Lucas Aloisi Guedes, Guilherme Dourado Zambon, Wilson Salgado

**Affiliations:** 1 Universidade de São Paulo – Medical School of Ribeirão Preto – Ribeirão Preto (SP), Brazil.

**Keywords:** Cholecystectomy, Risk Factors, Alanine Transaminase, Ultrasonography, Preoperative Care, Postoperative Complications

## Abstract

**Purpose:**

To identify preoperative factors predicting surgical difficulty in laparoscopic cholecystectomy at a secondary-level hospital.

**Methods:**

A retrospective study included 697 adults undergoing laparoscopic cholecystectomy from January 2021 to June 2024. Demographic, clinical, laboratory, and ultrasound data, as well as intra- and postoperative outcomes, were collected. Operative difficulty was graded using Nassar’s scale (I–V). Logistic regression analyses identified predictors of difficult cholecystectomy (Nassar III–V).

**Results:**

Among the 697 patients (81.5% female; mean age 46.7 ± 14.0 years old; mean body mass index 29.2 ± 4.8 kg/m^2^), 41.4% were classified as difficult. Conversion to open surgery occurred in 1.1%. Difficult cases showed longer operative time (79.9 ± 39.3 *versus* 56.9 ± 19.6 minutes, *p* 0.01), greater use of intraoperative cholangiography (12.5 *versus* 3.7%, *p* 0.01), longer postoperative stay (*p* 0.01), and higher incidence of nausea/vomiting (15.2 *versus* 7.8%, *p* 0.01). Multivariate analysis identified elevated alanine transaminase (*odds ratio* = 2.89, 95% confidence interval 1.80–4.64, *p* 0.001) and gallbladder wall thickening 4 mm (*odds ratio* = 4.75, 95% confidence interval 2.85–7.91, *p* 0.001) as independent predictors.

**Conclusion:**

Elevated alanine transaminase and gallbladder wall thickening are significant predictors of difficult laparoscopic cholecystectomy. Recognizing these factors may optimize surgical planning and enhance patient safety in non-tertiary hospitals.

## Introduction

Gallstone disease is highly prevalent, affecting roughly 10–15% of adults in the United States of America (U.S.) and up to 20% in Europe. It accounts for substantial morbidity and healthcare utilization, with recent U.S. data indicating about 2.2 million outpatient visits, 1.2 million emergency visits, 625,000 hospitalizations, and 2,000 deaths annually due to gallbladder disease^1^^,^ 2.

Cholecystectomy remains the definitive treatment for symptomatic cholelithiasis. Laparoscopic cholecystectomy (LC) quickly became the gold-standard approach, offering markedly lower overall morbidity (2–8%) and mortality (0.01%) compared to open surgery^3^.

Despite these benefits, a subset of LCs is technically difficult, often due to advanced inflammation or aberrant anatomy. Difficult LCs are associated with prolonged operative time, higher conversion rates to open surgery, greater postoperative complications, and extended length of stay^4^. Therefore, identifying high-risk cases preoperatively is crucial for patient counseling, operative planning, and patient safety^4^.

Numerous studies have sought to pinpoint risk factors for difficult LC and conversion. Established factors include acute cholecystitis (especially recurrent or severe episodes), cholangitis or choledocholithiasis, male sex, age over 60, obesity, prior upper abdominal surgery, and certain ultrasound findings such as gallbladder wall thickening, pericholecystic fluid, or an contracted (fibrosed) gallbladder^3^. Elevated inflammatory markers (*e.g.*, leukocytosis, C-reactive protein) and liver enzymes (*e.g.*, gamma-GT, alanine transaminase—ALT) have also been cited as potential predictors of difficult cholecystectomy and postoperative complications. Specifically, gallbladder wall thickness > 3–4 mm on ultrasonography is a consistently reported preoperative predictor of technical difficulty, conversion to open surgery, longer operative duration, and higher perioperative morbidity^4^. The presence of dense adhesions in the right upper quadrant (from prior inflammation or surgery) similarly portends a challenging procedure.

To synthesize these factors, various scoring systems have been proposed. Randhawa and Pujahari’s preoperative scoring method assigns points for clinical and ultrasound features to predict a “difficult LC”^5^. More recently, Nassar et al.^6^ formulated a comprehensive risk score using a validated intraoperative difficulty grading scale as an outcome. Such tools show promise, but their performance may vary with patient population and setting. For example, some scores validated in tertiary care centers with frequent acute cholecystitis cases might over-predict difficulty in secondary hospitals where the most severe cases are referred onward.

Our institution is a secondary-level hospital (medium complexity) which handles elective cholecystectomies but lacks on-site intensive care, interventional radiology, or advanced endoscopic services. Complex cases (*e.g.*, severe acute cholecystitis, suspected common bile duct stones requiring intraoperative clearance) are often triaged to higher-level centers, resulting in a somewhat filtered case mix. There is a need to understand which preoperative factors best predict surgical difficulty in this context, to optimize scheduling and decide when to refer patients preemptively to a tertiary facility. Large multi-center audits have characterized overall outcomes of cholecystectomy in various settings, including day-case surgery success rates and global practice variations during the COVID-19 pandemic^3^^–^ 5. However, data focusing on risk stratification in resource-limited hospitals remain scarce.

We aimed to identify key preoperative predictors of a difficult laparoscopic cholecystectomy in a secondary hospital environment and to compare our findings with published literature. We also evaluated how operative difficulty (as graded by Nassar’s scale) correlates with intra- and postoperative outcomes in this setting. Ultimately, the goal is to improve surgical planning and patient safety by recognizing high-risk cases before the operation, which is especially pertinent for hospitals without full tertiary support.

## Methods

### Study design and setting

We performed a retrospective observational study of patients who underwent LC at our hospital, from January 2021 to June 2024. This public university-affiliated hospital is a secondary-level facility primarily handling elective general surgeries. Notably, it has no on-site intensive care unit or blood bank, and it lacks the capability for intraoperative cholangiopancreatography or other advanced hepatobiliary interventions. Patients with very severe cholecystitis or complicated gallstone disease (*e.g.*, cholangitis, large common bile duct stones) are generally referred to tertiary centers. The study was approved by the Institutional Research Ethics Committee (CAAE: 6.942.455/2024 Clinics Hospital of Ribeirão Preto Medical School of the Universidade de São Paulo).

### Patients’ selection

We included all adult patients (≥ 18 years old) who underwent laparoscopic cholecystectomy for benign gallbladder disease (mostly symptomatic cholelithiasis and chronic or subacute cholecystitis). Elective outpatient cases were included, but patients who required upfront open cholecystectomy or who were transferred preoperatively to a higher-level hospital were excluded.

### Data collection

Using electronic medical records, we collected preoperative variables including demographic data (age, sex, body mass index, comorbidities), clinical presentation (symptoms, history of acute cholecystitis or biliary pancreatitis, prior abdominal surgeries), and laboratory results (white blood cell count, C-reactive protein, liver function tests such as alkaline phosphatase, gamma-glutamyl transferase, bilirubin, and ALT). Imaging findings from preoperative abdominal ultrasound were recorded, particularly gallbladder characteristics: presence of stones (number and size), gallbladder wall thickness (mm), presence of pericholecystic fluid collection, and bile duct dilatation or stones on imaging. We defined *elevated ALT* as any preoperative ALT value above the upper limit of normal for our lab (> 41 U/L). *Thickened gallbladder* wall on ultrasound was noted per the radiologist’s report (generally > 4-mm thickness). Randhawa’s preoperative score was calculated for each patient based on the method by Randhawa and Pujahari^5^. This score ranges from 0 to 15; in prior studies, a score ≥ 5 has been considered predictive of difficult cholecystectomy.

Intraoperative and postoperative data were also collected: the surgical approach (standard 4-port laparoscopy in all cases), any need to convert to open surgery, operative time (skin-to-skin, in minutes), whether an intraoperative cholangiogram was performed, and placement of drains. Intraoperative findings (*e.g.*, severe adhesions, gangrenous gallbladder) were noted from operative reports. Operative difficulty was graded according to Nassar’s scale, an objective scoring system from I (very easy) to V (very difficult) based on the visual and technical conditions encountered^6^.

For analysis, we defined *difficult cholecystectomy* as Nassar grade III, IV or V, corresponding to moderate to extreme difficulty requiring advanced technical effort. Postoperative outcomes recorded included: any complications within 30 days (using the Clavien-Dindo classification), length of postoperative hospital stay (hours or days), 30-day readmission, and whether the patient had fully recovered by the 30-day follow-up visit (defined in our service as being discharged from surgical outpatient care).

### Statistical analysis

Patients were stratified by operative difficulty (easy/moderate: Nassar I–II *versus* difficult: Nassar III–V) for comparative analysis. Categorical variables were compared using χ^2^ or Fisher’s exact test, and continuous variables with Student’s t-test or Mann-Whitney’s U test as appropriate. A univariate analysis was first performed to identify preoperative factors associated with difficult cases. Variables with *p* < 0.20 in univariate analysis were then entered into a multivariate logistic regression model (backward stepwise elimination) to determine independent predictors of a difficult LC. Adjusted *odds ratios* (OR) with 95% confidence intervals (95%CI) were calculated. Statistical significance was set at *p* < 0.05 (two-tailed). Results are presented as mean ± standard deviation or median (interquartile range) for continuous data, and proportions (%) for categorical data. Analyses were done using Statistical Package for the Social Sciences 27.0 (IBM Corp., Armonk, NY, United States of America).

## Results

### Patients’ characteristics

A total of 697 patients underwent LC during the study period and met inclusion criteria. The cohort was predominantly female (568 women, 129 men; 81.5% female). The mean age was 46.7 ± 14.0 years old, and the mean body mass index (BMI) was 29.2 ± 4.8 kg/m2, reflecting an overweight population. Most surgeries (94%) were elective or same-day admissions. Correspondingly, only 25 patients (3.6%) had an active acute cholecystitis at the time of surgery, whereas the majority had chronic calculous cholecystitis or biliary colic. A history of at least one prior gallstone-related emergency admission was noted in 17% of patients. About 12% had undergone previous abdominal surgery (mostly umbilical hernia repairs or gynecologic surgeries).

Laboratory tests showed elevated ALT in 19% of the patients preoperatively, while elevations in other liver enzymes or inflammatory markers were less common (for example, leukocytosis in 8%). On ultrasound, gallbladder wall thickening (> 4 mm) was present in 16% of the patients; ultrasound evidence of pericholecystic fluid in 6%; and a dilated common bile duct (> 6 mm) in 7%. Multiple gallstones (≥ 2) were seen in 54% of the cases and large (≥ 1 cm) gallstone in 22%. Using the Randhawa preoperative difficulty score, the mean score was 2.8 ± 1.4 points. Notably, only 29 patients (4.2%) had a Randhawa score > 5 (which would classify them as likely difficult by that method).

### Operative difficulty and outcomes

All procedures were initiated laparoscopically. The vast majority (689 cases, 98.9%) were completed successfully without conversion to open surgery. Eight patients (1.1%) required conversion to an open cholecystectomy due to inability to safely progress laparoscopically (reasons included severe adhesion obscuring Calot’s triangle in five cases and uncontrolled bleeding in three cases). According to the intraoperative Nassar grading, 408 patients (58.6%) were classified as easy (grade I or II), and 289 patients (41.4%) were classified as difficult. Difficult cases were associated with significantly prolonged operative times, need for additional intraoperative maneuvers, longer post-operative hospital stay, and more postoperative pain and nausea (Table 1).

**Table 1:** Surgical outcomes based on the Nassar’s intraoperative difficulty grading scale (Nassar 1 and 2: easier cholecystectomy; Nassar 3–5: more difficult).

		Nassar 1 and 2 (408)	Nassar 3–5 (289)	*p* -value
Duration of surgery (min)		56.92 ± 19.58	79.86 ± 39.25	<0.01
Intraoperative cholangiography (%)		15 (3.67)	36 (12.45)	< 0.01
Hospital stay (%)	≤ 24 h	372 (91.17)	238 (82.35)	< 0.01
	1–2 days	24 (5.88)	28 (9.69)	
	2–5 days	7 (1.71)	16 (5.53)	
	> 5 days	0 (0)	7 (2.42)	
	No information	5 (1.22)	0 (0)	
Extra medication in recovery (%)		45 (10.59)	34 (11.76)	0.62
Nausea and vomiting (%)		32 (7.84)	44 (15.22)	< 0.01
Fever (%)		0 (0)	2 (0.69)	NA
Bleeding (%)		1 (0.24)	6 (2.07)	NA
Pain (%)	Absent	309 (75.73)	198 (68.51)	0.03
	Mild	44 (10.78)	29 (10.03)	
	Moderate	27 (6.61)	36 (12.45)	
	Severe	28 (6.86)	26 (8.99)	
Cholestasis (%)		0 (0)	0 (0)	NA
Infection (%)		0 (0)	2 (0.69)	NA
Peritonitis (%)		0 (0)	2 (0.69)	NA
Reoperation (%)		0 (0)	2 (0.69)	NA

NA: no analysis.

Source: Elaborated by the authors.

Placement of a subhepatic drain at the end of the procedure was also more frequent in the difficult group (30 *versus* 10%, *p* < 0.001). No bile duct injuries occurred in either group. Two patients in the difficult cohort required intraoperative partial cholecystectomy (fundus-first “subtotal” technique) due to inability to safely dissect Calot’s triangle – both were counted as grade-V difficulty and recovered without major complication.

There were two cases of postoperative bile leak in the difficult group (both managed conservatively with drainage), and none in the easy group. There were no 30-day mortalities in either group. By the time of the routine 30-day follow-up clinic visit, 26.1% of difficult-case patients had not been “discharged” from care yet (meaning they required ongoing outpatient follow-up or further intervention), compared to 16.1% of the easier-case patients. In other words, only 66.3% of patients in the difficult group achieved an uncomplicated recovery by 30 days versus 73.9% in the easy group (p = 0.03).

### Univariate analysis of predictors

On univariate testing, several factors were significantly associated with a higher likelihood of difficult surgery (Nassar III–V). These included: history of acute cholecystitis (32 *versus* 10%, *p* < 0.001), presence of nausea/vomiting as a presenting symptom (45 *versus* 20%, *p* < 0.001), and any indication of cholestasis (such as jaundice or elevated cholestatic liver enzymes; 18 *versus* 5%, *p* < 0.001). Among laboratory tests, ALT elevation stood out (35% of difficult cases had ALT above normal *versus* 15% of easy cases, *p* < 0.001). Elevation of gamma-GT and alkaline phosphatase showed a similar trend but overlapped with ALT in many cases of suspected choledocholithiasis. Inflammatory markers like elevated leukocyte count and C-reactive protein > 10 mg/L were more frequent in difficult cases (25 versus 6%, and 30 *versus* 8% respectively, both *p* < 0.001). Ultrasonographic findings significantly associated with difficulty were gallbladder wall thickening (> 4 mm in 44% of difficult cases *versus* 8% of easy cases, *p* < 0.001) and pericholecystic fluid (10 *versus* 2%, *p* = 0.002). A dilated common bile duct on imaging was also more common in the difficult group (15 *versus* 4%, *p* < 0.01), often indicating possible passed or retained stones. Patient-related factors like age > 60, male sex, BMI ≥ 30, and diabetes were observed more often in difficult cases but did not reach statistical significance in our cohort. Prior upper abdominal surgery showed a trend toward more difficulty (5 *versus* 2%, *p* = 0.09). The preoperative Randhawa score was higher on average in difficult cases (mean 3.5 *versus* 2.5, *p* < 0.01), and a Randhawa score > 5 was found in 18 of the 196 difficult cases (9.2%) compared to 11 of 502 easy cases (2.2%)—this difference was significant (*p* < 0.01). Thus, the Randhawa scoring system identified some high-risk patients, but many difficult cases had low-intermediate scores and many high scores corresponded to only moderately difficult surgeries, resulting in a weak overall correlation (Cramer’s V = 0.07).

### Multivariate analysis

All variables with *p* < 0.20 on univariate screening were entered into a logistic regression. After adjusting for inter-correlations (for instance, acute presentation correlating with ultrasound findings of inflammation), the model retained two independent predictors of a difficult LC: elevated ALT and gallbladder wall thickening on ultrasound. The presence of a preoperative ALT above normal carried an adjusted OR of 2.89 (95%CI 1.80–4.64, *p* < 0.001) for Nassar grade III–V difficulty. Gallbladder wall thickening > 4 mm had an OR of 4.75 (95%CI 2.85–7.91, *p* < 0.001). These two factors remained highly significant after controlling for all other factors. In contrast, other variables, such as emergency admission, prior acute cholecystitis, and abnormal biliary imaging, did not maintain independent significance in the multivariate model. The Randhawa preoperative score, when included as a composite variable, was not a significant independent predictor in the multivariate model (likely due to its components being represented by the above specific factors). The final model had a c-statistic of 0.81, indicating good discriminative ability.

## Discussion

In this study of nearly 700 patients at a secondary-level hospital, we found that preoperative ALT elevation and gallbladder wall thickening on ultrasound are the strongest independent predictors of increased surgical difficulty in laparoscopic cholecystectomy. These findings add to the body of evidence on risk stratification in LC, while highlighting considerations unique to a non-tertiary care setting. Overall, our difficult-case rate (28%) and conversion rate (1.1%) are consistent with reports from the literature, although direct comparisons must account for differences in case mix. Population-based series have noted conversion rates around 2.9–3.2% for elective LC in general practice, with higher rates in older, sicker populations, and during acute cholecystitis^3^. Our low conversion rate likely reflects the practice of referring the most severe cases to higher centers, as well as the high proportion of elective (non-acute) surgeries (over 90%).

In our hospital, only a small subset of cholecystectomies was done for acute cholecystitis, and they contributed heavily to the *difficult* category. It is notable that even in an ambulatory surgery-oriented center, over a quarter of LCs had significant difficulty by the Nassar grading. This underscores that challenging gallbladder pathology is not exclusive to tertiary hospitals; secondary hospitals will inevitably encounter difficult cases and should prepare accordingly.

Our results concur with prior studies identifying gallbladder wall thickness as a key predictor of difficult LC7. A thickened wall typically indicates chronic inflammation or fibrosis from repeated cholecystitis, which correlates with dense adhesions and obscured anatomy intraoperatively. Kokoroskos et al.^8^ reported in a prospective series of 1,089 patients that wall thickness > 4 mm significantly predicted longer operative times, higher blood loss, and need for advanced techniques. Similarly, Chand et al.9 found that ultrasound features like increased wall thickness and pericholecystic fluid were associated with higher difficulty levels in LC. Our multivariate analysis confirms wall thickness as an independent factor, with an almost five-fold increased odds of a difficult surgery. This suggests that a simple preoperative ultrasound measurement can be a powerful tool in risk stratification. It also reinforces the recommendation that surgeons carefully review gallbladder ultrasound findings prior to LC; even in routine cases, the presence of a thick-walled gallbladder should alert the team to potential challenges such as difficult dissection or the need for subtotal cholecystectomy.

The association of elevated ALT with difficult cholecystectomy is another notable finding of our study. ALT is a liver enzyme that typically rises in hepatocellular injury but can also be elevated in acute biliary obstruction or inflammation (often along with other enzymes). In our context, many patients with raised ALT had either passed small stones through the common bile duct or had ongoing cholestatic stress on the liver. We postulate that elevated ALT serves as a surrogate for more severe biliary pathology, such as transient obstruction of the cystic or common bile duct or even concomitant cholangitis. These conditions lead to inflamed and edematous tissue planes, making dissection more arduous.

Other authors have similarly observed that laboratory markers of inflammation or cholestasis, including transaminases, are frequently higher in cases that turn out to be difficult LCs or require conversion^8^. While ALT itself may not directly cause difficulty, it flags underlying pathology that does. Importantly, our finding regarding ALT remained significant even when controlling for clinical diagnosis of cholangitis or ultrasound evidence of common bile duct stones, suggesting that even mild liver enzyme elevations (which might be overlooked clinically) should raise caution. It may be prudent, in secondary hospitals, to investigate the cause of ALT elevation before proceeding to surgery.

Interestingly, classic risk factors like male sex, older age, high BMI, and diabetes were not independent predictors in our adjusted analysis, even though they have been reported in larger series^3^. This could be due to the composition of our patient sample or the referral pattern. Our cohort’s mean age was relatively young (mid-40s) and only 3% were ASA III or above; extremely elderly or frail patients with gallstones may have been managed in other hospitals. Male sex showed a trend toward more difficulty (as it has often been noted anecdotally, possibly due to delayed healthcare seeking leading to larger stones or more fibrosis in men), but in our data it did not reach significance^3^. Similarly, while obesity can make laparoscopy challenging, our surgeons are experienced with high-BMI patients, and the BMI difference between groups was modest (the average patient was overweight in both groups).

Our evaluation of the Randhawa and Pujahari preoperative scoring system in this cohort yielded mixed results. The score correlated with surgical difficulty to a degree—patients with higher scores were more likely to have difficult surgeries—, but its predictive accuracy was limited. Only 4% of our patients exceeded the threshold score of 5, reflecting the lower prevalence of extreme risk features in our setting (*e.g.*, very few patients had *all* risk factors concurrently). Consequently, the score had low sensitivity; many difficult cases had intermediate scores rather than high. We also found no significant correlation between Randhawa’s score and the Nassar intraoperative grade (Spearman *p* ≈ 0). This contrasts with some studies in tertiary hospitals where preoperative scores have performed better. For instance, Gupta et al.^10^ reported a good correlation of Randhawa’s score with operative difficulty in a high-volume center dealing with acute cholecystitis, suggesting the score’s utility may be context dependent. In our medium-complexity hospital, the scarcity of high-scoring patients and the referral of very severe cases likely blunted the score’s effectiveness. This finding is in line with our hypothesis that existing scoring systems may need adaptation for different care levels. A score developed in a setting with frequent severe disease may over-predict difficulty in a filtered population. We advocate caution in applying generic difficulty scores universally; local calibration might be necessary. It is notable that Nassar’s own group developed an extensive risk score in 2020 incorporating numerous variables to predict difficult LC. While comprehensive, its practicality at the bedside remains to be proven, and simpler proxies (like ALT and wall thickness) might suffice in many cases.

The clinical implications of our findings are particularly relevant for secondary hospitals and ambulatory surgery centers. First, recognizing ALT elevation and gallbladder wall thickening as red flags allows for better planning: a patient with these features might be scheduled on a day when senior surgical staff are available, rather than with a trainee alone, and when conversion or longer operative time can be accommodated. If both factors are present together, the risk of difficulty is especially high—our data suggest these patients often had the most complex courses. Combining predictors has been suggested as a strategy; for example, one might consider that a patient who simultaneously has abnormal ALTs and a thick-walled gallbladder on ultrasound is an ideal candidate for referral to a higher center or at least a very cautious approach. In our study, the coexistence of elevated ALT and wall thickening was relatively rare, but it portended almost certain difficult dissection.

Secondly, for hospitals lacking tertiary support (intensive care unit, endoscopic retrograde cholangiopancreatography, interventional radiology), the threshold for referral or for performing a planned open cholecystectomy should be low when high-risk features are identified. Our results support a strategy of selective referral: for instance, a patient with suspected cholangitis or very inflamed gallbladder (indicated by high ALT and ultrasound changes) could benefit from being managed in a center where intraoperative cholangiography and bile duct exploration are readily available, rather than risking an incomplete procedure or complications in a low-resource setting. While our surgeons managed to avoid any bile duct injuries and kept conversion rates low, this was likely aided by careful case selection and a willingness to convert early or perform subtotal cholecystectomy when needed. These decisions were made preemptively in some cases—for example, two patients with wall thickness > 10 mm and very high liver enzymes were planned for a subtotal approach from the outset. This kind of proactive surgical planning can be informed by the predictors identified in this study.

Our analysis also highlights that difficult cases have measurably worse perioperative outcomes even when managed laparoscopically. We observed longer operative times and hospital stays, as well as a higher incidence of postoperative nausea, likely related to longer anesthesia and greater manipulation. These findings agree with prior research such as the CholeS national audit^11^, which found that prolonged surgeries and multiple emergency admissions pre-operatively were associated with higher 30-day complication and readmission rates. Nassar’s difficulty grading in our study strongly stratified outcomes: patients with Nassar grade ≥ 3 had significantly more postoperative issues, paralleling Nassar et al.’s own report in which increasing grades correlated with escalating morbidity^6^. This reinforces the concept that identifying difficult cases is not merely academic, but directly relevant to patient prognosis. It begs the question of whether such patients should receive enhanced postoperative monitoring or interventions (for example, routine overnight observation rather than day-case discharge, as indeed happened in our practice).

### Limitations

The retrospective design of our study imposes certain limitations. The accuracy of predictor variables was dependent on documentation; for instance, symptom duration was not reliably recorded for many patients, so we could not analyze whether a long history of biliary colic predicted difficulty. There is also an inherent selection bias, as the most severe gallstone cases never entered our dataset (having been referred out). This filtering likely reduced the incidence of extreme presentations (*e.g.*, Mirizzi syndrome, severe acute cholecystitis with sepsis) that would be difficult. Consequently, our findings might underestimate the full spectrum of risk factors that would be seen in an unfiltered population. Conversely, it allowed us to pinpoint subtler predictors (like mild enzyme elevations) that might be overshadowed in a sicker cohort. Another limitation is the low number of events for some outcomes (*e.g.*, zero bile duct injuries, few conversions), which prevented meaningful statistical analysis of predictors for those specific endpoints. Additionally, our definition of difficult surgery was tied to the Nassar’s grading, which, while objective, has a subjective component and might vary slightly between surgeons.

## Conclusion

Operative difficulty in LC is associated with worse clinical outcomes, even in an ambulatory surgical setting. In our secondary hospital study, the intraoperative Nassar’s grading proved to be a useful tool to stratify cases by risk, correlating with operative time and postoperative recovery. More importantly, we identified elevated ALT and ultrasound-detected gallbladder wall thickening as independent preoperative predictors of a difficult cholecystectomy. These objective factors can be readily obtained during routine evaluation and should alert surgeons to potential technical challenges.

In practice, incorporating these predictors into surgical planning can improve patient safety and efficiency. Patients with high-risk features may benefit from being scheduled with experienced surgeons, having a low threshold for conversion or subtotal cholecystectomy, or being referred to higher-level centers if appropriate infrastructure (*e.g.*, intensive care unit, ERCP) might be needed.

Optimizing surgical planning based on objective data can lead to better outcomes, ensuring that even hospitals of medium complexity can perform LC with high success rates and minimal complications. Ultimately, prospective studies and the development of tailored risk scores could further enhance our ability to predict and manage difficult gallbladder cases in diverse healthcare settings.

## Data Availability

The data will be available upon request.
